# High expression of GPR116 indicates poor survival outcome and promotes tumor progression in colorectal carcinoma

**DOI:** 10.18632/oncotarget.18203

**Published:** 2017-05-25

**Authors:** Li Yang, Xiao-Lu Lin, Wei Liang, Seng-Wang Fu, Wen-Feng Lin, Xiao-Qing Tian, Yun-Jie Gao, Hao-Yan Chen, Jun Dai, Zhi-Zheng Ge

**Affiliations:** ^1^ Division of Gastroenterology and Hepatology, Key Laboratory of Gastroenterology and Hepatology, Ministry of Health, Renji Hospital, School of Medicine, Shanghai Jiao Tong University, Shanghai Institute of Digestive Disease, Shanghai 200001, China; ^2^ Division of Gastroenterology and Hepatology, Key Laboratory of Gastroenterology and Hepatology, Ministry of Health, State Key Laboratory for Oncogenes and Related Genes, Renji Hospital, School of Medicine, Shanghai Jiao Tong University, Shanghai Institute of Digestive Disease, Shanghai 200001, China; ^3^ Department of Digestive Endoscopy, Provincial Clinic Medical College, Fujian Medical University, Fujian Provincial Hospital, Fuzhou 350001, China; ^4^ Department of Gastroenterology, Shanghai General Hospital, Shanghai Jiao Tong University School of Medicine, Shanghai 200080, China

**Keywords:** GPR116, prognosis, metastasis, colorectal carcinoma

## Abstract

Previous studies have found that G-protein-coupled receptor 116 (GPR116) is a regulator of breast cancer metastasis. However, the role of GPR116 in colorectal carcinoma (CRC) carcinogenesis and progression is unknown. In this study, We found GPR116 expression was significantly up-regulated in CRC specimens compared with corresponding non-cancerous tissues. Increased GPR116 expression in CRC was correlated with histological differentiation and distant metastasis. In addition, high expression of GPR116 was significantly associated with poor overall survival of CRC patients, which was also confirmed by GSE14333, GSE17536 and GSE33113 datasets from the Gene Expression Omnibus (GEO). Furthermore, we demonstrated that the ability of proliferation and invasion of CRC cell lines HCT116 and LOVO was markedly reduced after transfected with siRNA-GPR116. Meanwhile, GPR116 may drive EMT in CRC cells through AKT/EKR signaling pathway, resulting in metastasis. Thus, GPR116 may be a novel reliable prognostic indicator and a risk factor in CRC progression.

## INTRODUCTION

Colorectal carcinoma (CRC) is the third most common cancer and the fourth leading cause of cancer-related mortality in the world [1, 2]. Despite the introduction of early diagnosis and treatment options in the past few years, the prognosis of CRC patients is still not satisfactory. Due to the high rate of recurrence and distant metastasis, the 5-year survival rate of CRC is less than 50% in low-income countries [3–5]. Although clinical and pathological features play a major role in determining the treatment approach of CRC, the prognosis of CRC patients after surgical resection varies greatly, even when patients are assigned to the same TNM stage [6]. Therefore, identification of a new biomarker and an effective prognostic predictor is urgent in the early diagnosis, evaluation of prognosis and personalized treatment of CRC.

G-protein-coupled receptors (GPCRs) are the largest family of proteins in the cell membrane, and they are involved in various pathophysiological processes of organisms. Based on the human G-protein-coupled receptors families (GRAFS) classification, GPCRs have five sub-types, including glutamate, rhodopsin, adhesion, frizzled/taste and secretin [7–9]. Among them, the adhesion sub-type has long extracellular components with a number of structural domains to facilitate cell and extracellular matrix interactions. Vitro studies demonstrate that the adhesion sub-type plays role in cell motility, migration, and adhesion [10–15]. In addition, an increasing amount of evidence demonstrates the role of the adhesion sub-type in tumor cell metastasis [16].

GPR116 is a member of the adhesion sub-type of GPCRs family, which is composed of 33 members in humans with a variety of distribution in embryonic cells, reproductive tract cells, leukocytes, neurons, and tumor cells [17–18]. Tang et al. first found that GPR116 may be strongly correlated with breast cancer stage, metastasis, and progression through the Gaq-p63RhoGEF-Rho GTPase signaling pathway [19]. However, little is known about the role of GPR116 in the carcinogenesis and progression of colorectal carcinoma.

This study aimed to assess the expression pattern of GPR116 in human CRC, examine the relationship of GPR116 expression with clinical and pathological parameters in CRC patients and determine the role of GPR116 in CRC progression.

## RESULTS

### The expression of GPR116 is significantly up-regulated in CRC

**Figure 1:**
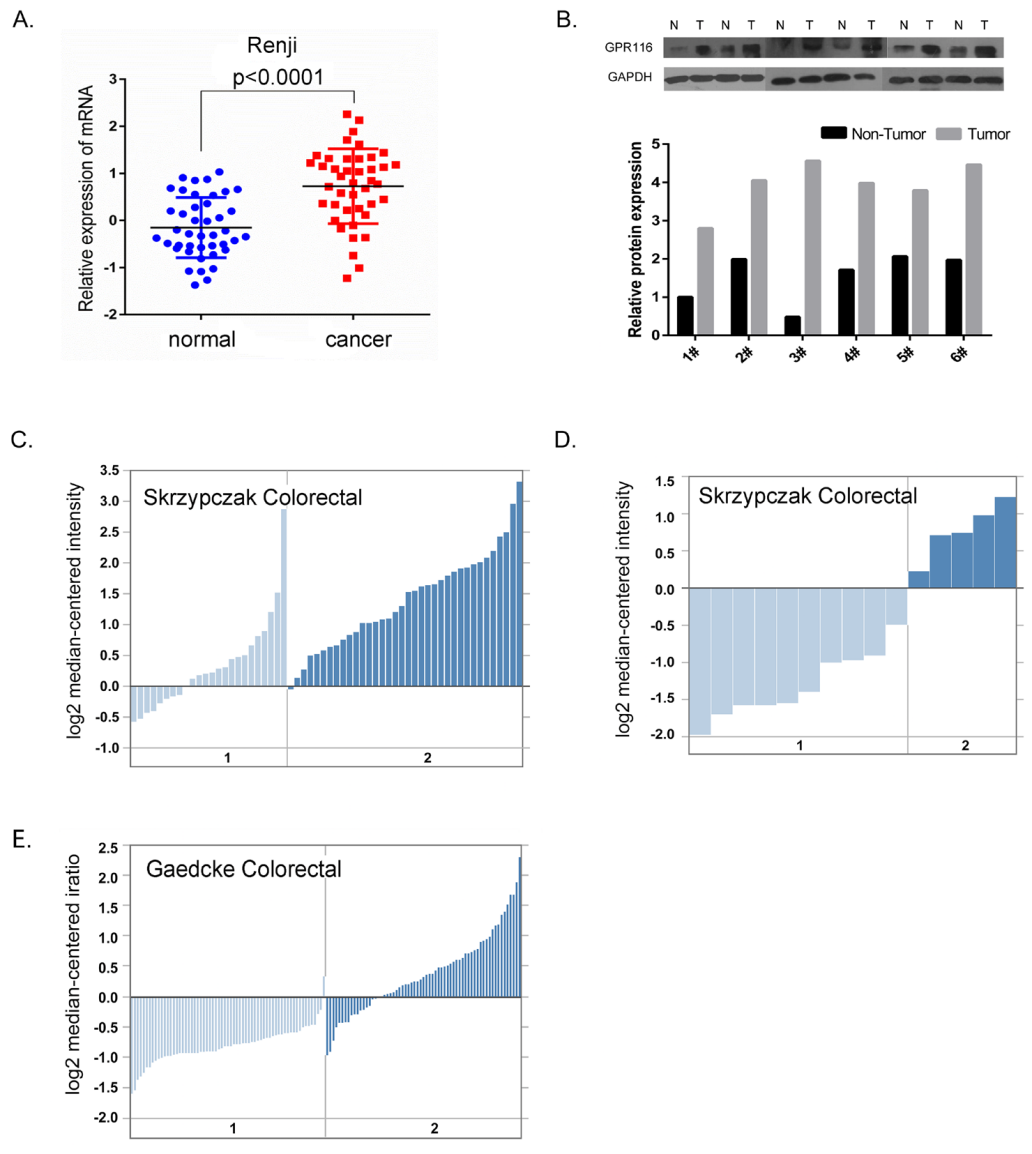
GPR116 expression was increased in colorectal carcinoma at the mRNA and protein level **(A)** The mRNA expression level of GPR116 in colon cancer and matched normal colon tissue derived from the Renji cohort was detected by real-time quantitative PCR. Error bars in the scatter plots represent SE. **(B)** Western blots showed GPR116 protein level in six paired colon cancer tissues (T) and adjacent normal colonic tissue (N) of patients from the Renji cohort and the relative GPR116 protein expression. GPR116 expression in Skrzypczak Colorectal **(C)** Skrzypczak Colorectal 2 **(D)** and Gaedcke Colorectal **(E)** grouped by normal colorectal tissue (1) and colorectal carcinoma (2) in Oncomine Cancer Microarray database. CRC: colorectal carcinoma; GPR116: G protein-coupled receptor 116; GAPDH: glyceraldehyde-3-phosphate dehydrogenase; SE: standard error.

We firstly performed RT-PCR and western blot to assess the expression level of GPR116 in 48 cases of CRC tissues and matched normal tissues (Figure 1A-1B). As expected, GPR116 was overexpressed in CRC tissues compared with their non-cancerous counterparts at both the mRNA and protein levels. In addition, three independent microarray datasets from the Oncomine database were extracted to analyze GPR116 expression (Figure 1C-1E). According to the database, the majority of GPR116 mRNA expression was remarkably higher in cancer specimens compared with the adjacent non-cancerous tissues, which was consistent with our results.

**Figure 2:**
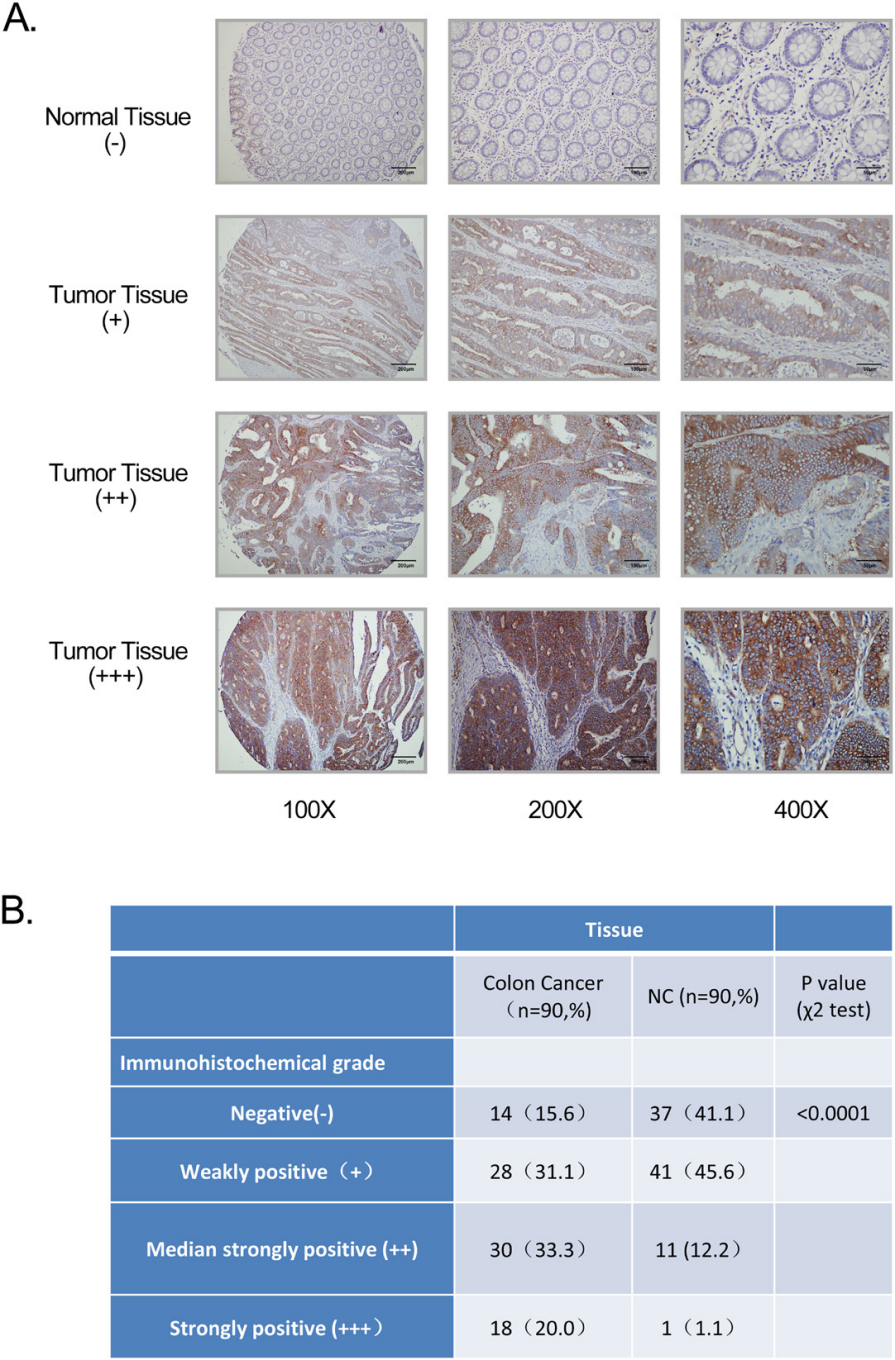
GPR116 expression in colon cancer tissue samples **(A)** GPR116 protein level was measured by immunohistochemical analysis in normal colon and colon cancer tissue with different staining intensities. Original magnification ×100 (bar=200μm); 200 (bar=100μm); 400 (bar=50μm). **(B)** The numbers of different immunohistochemical grade expression in normal colon and colon cancer tissue. NC: normal colon.

To explore the clinical significance of GPR116 overexpression, immunohistochemical (IHC) staining was used to determine GPR116 protein expression in 90 colorectal carcinoma samples. According to the IHC scoring criteria in the method, 48 of 90 (53.3%) CRC specimens showed high GPR116 expression (GPR116 ++ or GPR116 +++), whereas the remaining 42 CRC samples (46.7%) displayed low GPR116 expression (GPR116- or GPR116 +) (Figure 2A-2B). The tissue microarrays analysis also confirmed that GPR116 protein displayed higher expression in tumor tissues than adjacent normal tissues (Figure 2B) p<0.0001).

### Clinical and pathologic features of patients with CRC

The 90 CRC patients included 38 females and 52 males, with a median age of 67.14±10.73 years. Our study included 5 cases of stage I CRC, 37 cases of stage II CRC, 43 cases of stage III CRC, and 5 cases of stage IV CRC with liver metastasis. The histological grades of these patients included 56 patients with well/moderately differentiated tumors and 34 patients with poorly differentiated tumors (Table 1). The histological grades and clinical stages were determined based on the tumor-node-metastasis (TNM) staging system for CRC defined by the American Joint Committee on Cancer.

**Table 1:** Association between GPR116 expression and clinicopathologic features in patients with colorectal carcinoma

Characteristics	Total(n=90)	GPR116 expression		*P* value(χ^2^ test)
		High (n=48)	Low (n=42)	
Age (years)				
Mean (years)				
<65	35	19 (54.3)	16 (45.7)	0.885
≥65	55	29 (52.7)	26 (47.3)	
Gender				
Male	52	28 (53.8)	24 (46.2)	0.909
Female	38	20 (52.6)	18 (47.4)	
Tumor location				
Ascending	28	17 (60.7)	11 (39.3)	0.522
Transverse	20	9 (45.0)	11 (55.0)	
Descending	16	10 (62.5)	6 (37.5)	
Sigmoid	26	12 (46.2)	14 (53.8)	
TNM stage (AJCC)				
Stage I	5	0 (0.0)	5 (100.0)	0.102
Stage II	37	20 (54.1)	17 (45.9)	
Stage III	43	25 (58.1)	18 (41.9)	
Stage IV	5	3 (60.0)	2 (40.0)	
Size				
<5cm	34	16 (47.1)	18 (52.9)	0.353
≥5cm	56	32 (57.1)	24 (42.9)	
Histological differentiation				
Well/moderate	56	25 (45.5)	31 (54.5)	**0.034**
Poor	34	23 (65.7)	11 (34.3)	
Lymph node metastasis				
Absent	46	22 (47.8)	24 (52.2)	0.284
Present	44	26 (59.1)	18 (40.9)	
Distant metastasis				
Absent	85	43 (50.6)	42 (49.4)	**0.031**
Present	5	5 (100.0)	0 (0.0)	

Values in parentheses indicate percentage values. The bold number represents the *P*-values with significant differences.

### Correlation of GPR116 expression with clinicopathological characteristics and prognosis in CRC patients

We used the Chi-square test or Fisher's exact test to evaluate the correlation between GPR116 expression and corresponding patients’ clinicopathological parameters. The results indicated that GPR116 expression in CRC specimens was significantly correlated with histological differentiation (*p*=0.034) and distant metastasis (*p*=0.031), whereas age, gender, tumor location, TNM stage, tumor size and lymph node metastasis had no significant relationship with GPR116 expression (Table 1).

**Figure 3:**
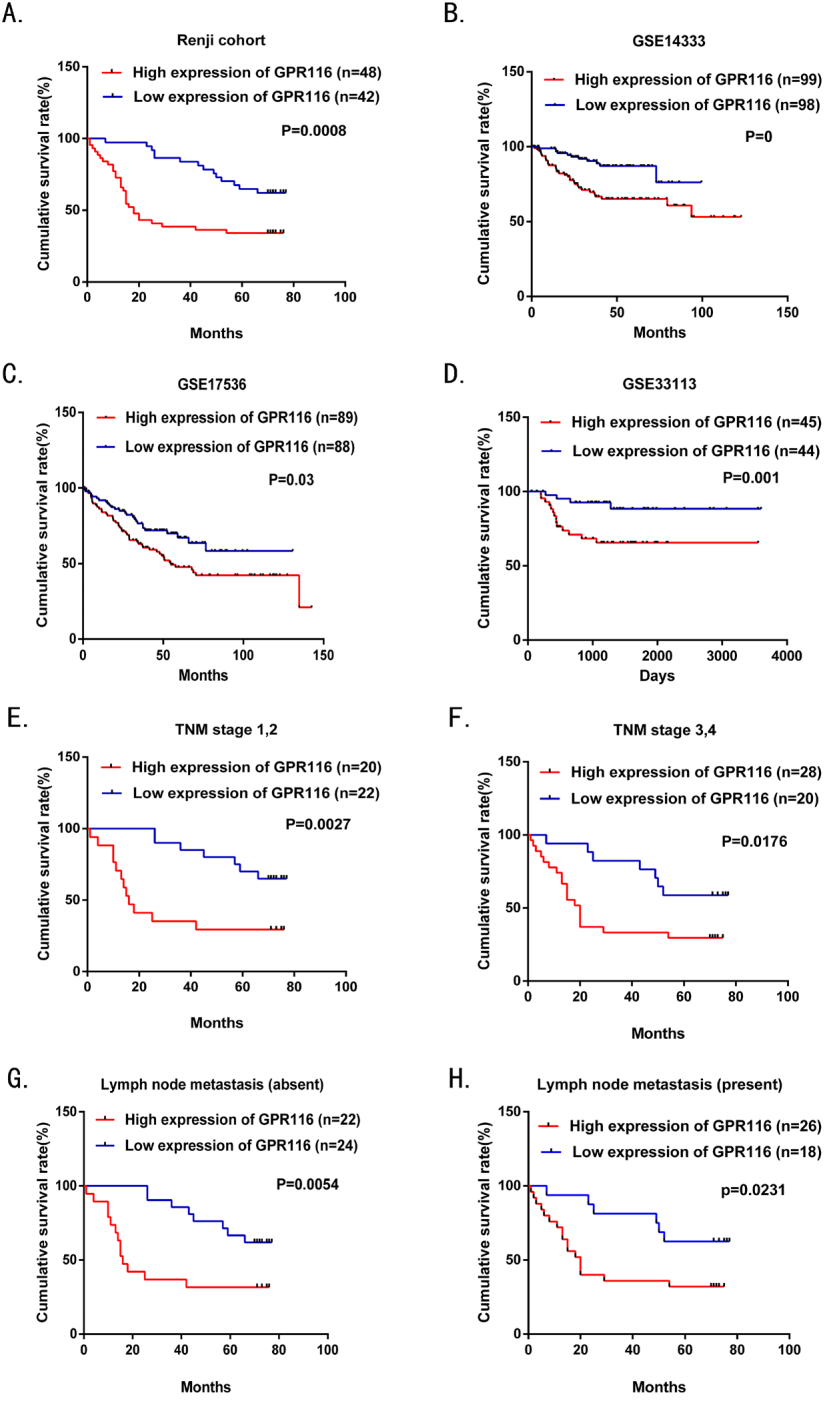
Overall survival analysis of CRC patients with different GPR116 protein expression **(A)** Overall survival analysis of 90 CRC patients with different GPR116 protein expression in the Renji cohort. **(B-D)** The association of GPR116 expression and patient survival was conducted in GSE14333, GSE17536, and GSE33113 datasets, respectively. **(E-F)** Comparisons of overall survival between the lower GPR116 expression group and the higher GPR116 expression group in the early TNM stage (I-II) cohort and in the advanced TNM stage (III-IV) cohort. **(G-H)** Comparisons of overall survival between the lower GPR116 expression group and the higher GPR116 expression group in patients with or without lymph node metastasis. *p*-values were calculated by Log-rank test. TNM: tumor-node –metastasis.

To evaluate the prognostic role of GPR116 in CRC patients, the correlations between GPR116 expression and corresponding clinical parameters were analyzed by Log-rank test and Kaplan–Meier analysis. The survival times of GPR116 high expression and low expression group were 34.59±29.49 and 61.51±19.70 months respectively. The survival time was significantly shorter in patients with higher GPR116 expression than those with lower GPR116 expression (Figure 3A, *p*=0.0008). To increase the reliability of this result, we obtained the prognostic significance of GPR116 in GSE14333, GSE17536, and GSE33113 with a total of 197, 177, and 89 cases enrolled from the GEO datasets. We found that high GPR116 protein expression was obviously associated with reduced overall survival time (*p*=0, 0.03, and 0.001 respectively, Figure 3B-3D). Furthermore, we assessed the correlation between GPR116 expression and overall survival time in CRC patients in early or advanced TNM stage as well as in the presence or absence of lymphatic metastasis. Our research demonstrated that the overall survival time was decreased in CRC patients with higher GPR116 expression regardless of TNM stage (Figure 3E-3F) and lymphatic metastasis by using Kaplan–Meier analysis (Figure 3G-3H).

**Figure 4:**
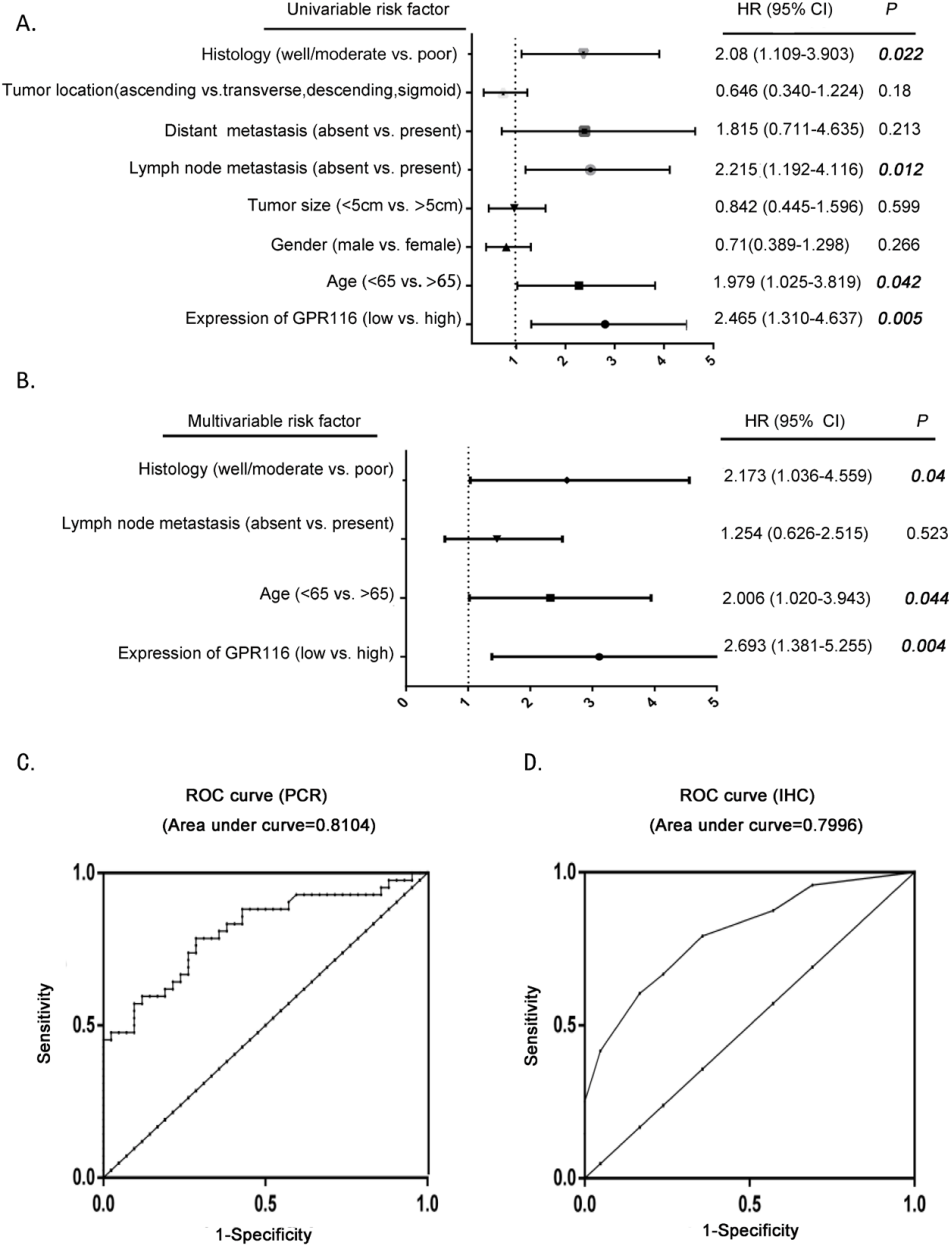
The potential value of GPR116 expression in predicting CRC and patient prognosis **(A-B)** The forest plot showed the correlation between CRC patient overall survival and GPR116 expression as well as other clinical characteristics by using univariable and multivariable analysis. **(C)** ROC curve analysis according to GPR116 expression by RT-PCR. **(D)** ROC curve analysis according to GPR116 expression by IHC. ROC: receiver operating characteristic; IHC: immunohistochemical.

To investigate whether GPR116 expression was an independent prognostic parameter in CRC, we used the Cox regression analysis to determine the effect of each variable on survival. Univariate analysis showed that GPR116 expression, age, lymph node metastasis, and histology were markedly associated with disease-specific survival (Table 2, Figure 4A). Multivariate analysis found that GPR116 expression, age and histology were three independent prognostic indicators for patients with CRC (Table 2, Figure 4B). Furthermore, receiver operating characteristic curve analyses were conducted to investigate the prediction value of GPR116 in CRC according to its mRNA expression and immunohistochemical scoring. Results demonstrated that GPR116 was a practical predictor, with an area under curve of 0.8104 and 0.7996 (Figure 4C-4D). All of these results indicated that high expression of GPR116 may predict unfavorable prognosis in CRC patients.

**Table 2:** Univariate and multivariate analysis of prognostic parameters for survival in patients with colorectal carcinoma

Prognostic parameter	Univariate analysis			Multivariate analysis		
	HR	95% CI	*P* value	HR	95% CI	*P* value
Expression of GPR116 (low vs. high)	2.465	1.310-4.637	**0.005**	2.693	1.381-5.255	**0.004**
Age (<65 vs. ≥65)	1.979	1.025-3.819	**0.042**	2.006	1.020-3.943	**0.044**
Gender (male vs. female)	0.710	0.389-1.298	0.266	_	_	_
Tumor size (<5cm vs. ≥5cm)	0.842	0.445-1.596	0.599	_	_	_
Lymph node metastasis (absent vs. present)	2.215	1.192-4.116	**0.012**	1.254	0.626-2.515	0.523
Distant metastasis (absent vs. present)	1.815	0.711-4.635	0.213	_	_	_
Tumor location (ascending vs. transverse, descending, sigmoid)	0.646	0.340-1.224	0.180	_	_	_
Histology (well/moderate vs. poor)	2.080	1.109-3.903	**0.022**	2.173	1.036-4.559	**0.040**

HR: hazard ratio; CI: confidence interval. The bold number represents the *P*-values with significant differences.

### Biological functions of GPR116 in CRC

**Figure 5:**
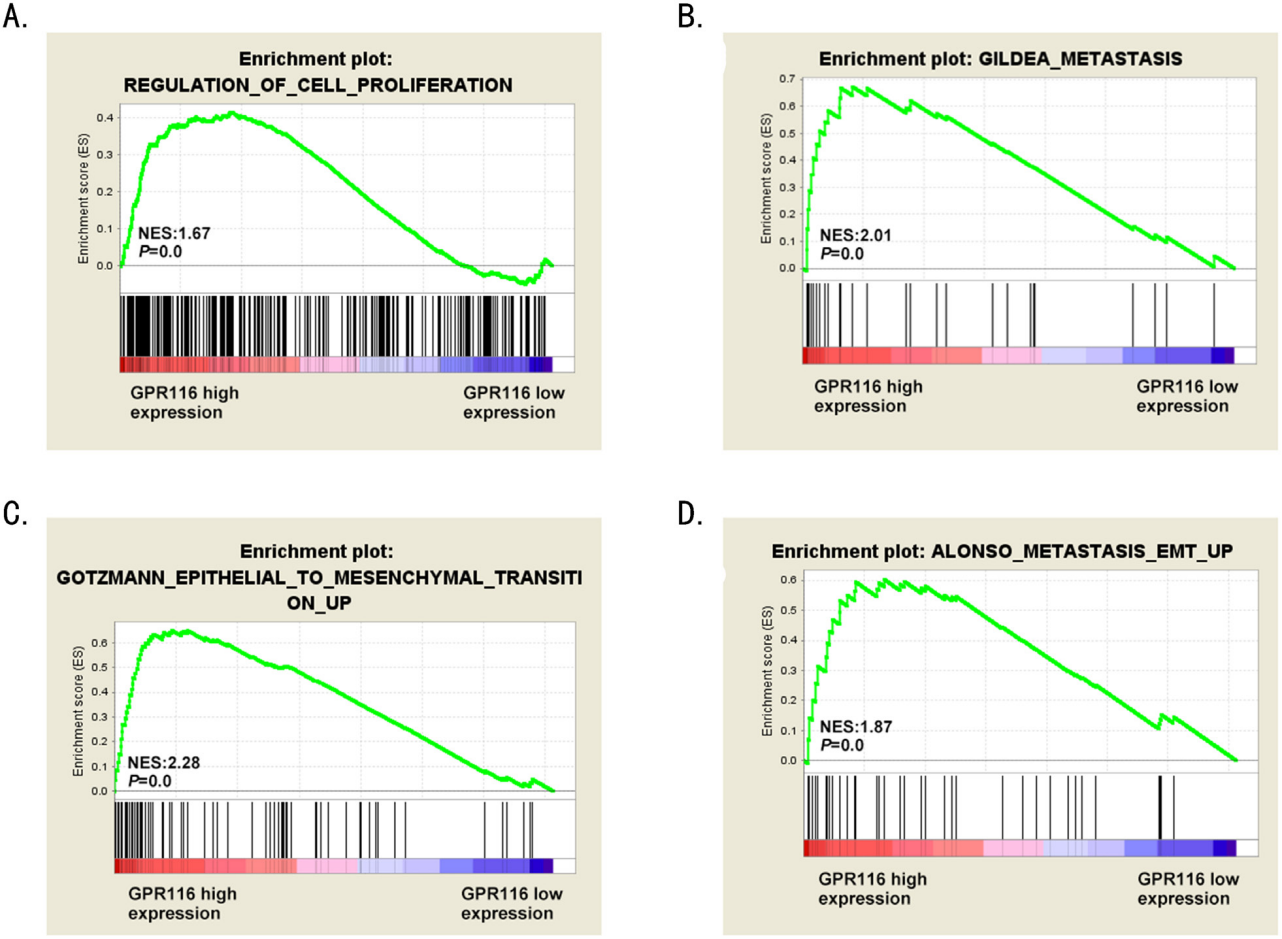
The biological functions of GPR116 in CRC **(A-D)** GSEA revealed that “Regulation_Of_Cell_Proliferation”, “Gildea_Metastasis”, “Gotzmann_Epithelial_ To_Mesenchymal_Transiton_Up” and “Alonso_Metastasis_ EMT_Up” pathway were enriched in GPR116-high expression group compared with GPR116-low expression group. The enrichment score (ES, green line) means the degree to which the gene set is overrepresented at the top or bottom of the ranked list of genes. EMT: epithelial-mesenchymal transition.

Gene set enrichment analysis (GSEA) was performed in The Cancer Genome Atlas (TCGA) datasets to acquire the biologic functions involved in CRC pathogenesis stratified by the median of GPR116 expression level. According to the enrichment plots of GSEA, the gene signatures of cell proliferation, metastasis and epithelial to mesenchymal transition (EMT) were more active in patients with higher GPR116 expression (Figure 5A-5D). Thus, we could raise hypotheses that overexpression of GPR116 may be involved in CRC progression.

### Knockdown of GPR116 decreases the proliferation and invasion ability of CRC cells

**Figure 6:**
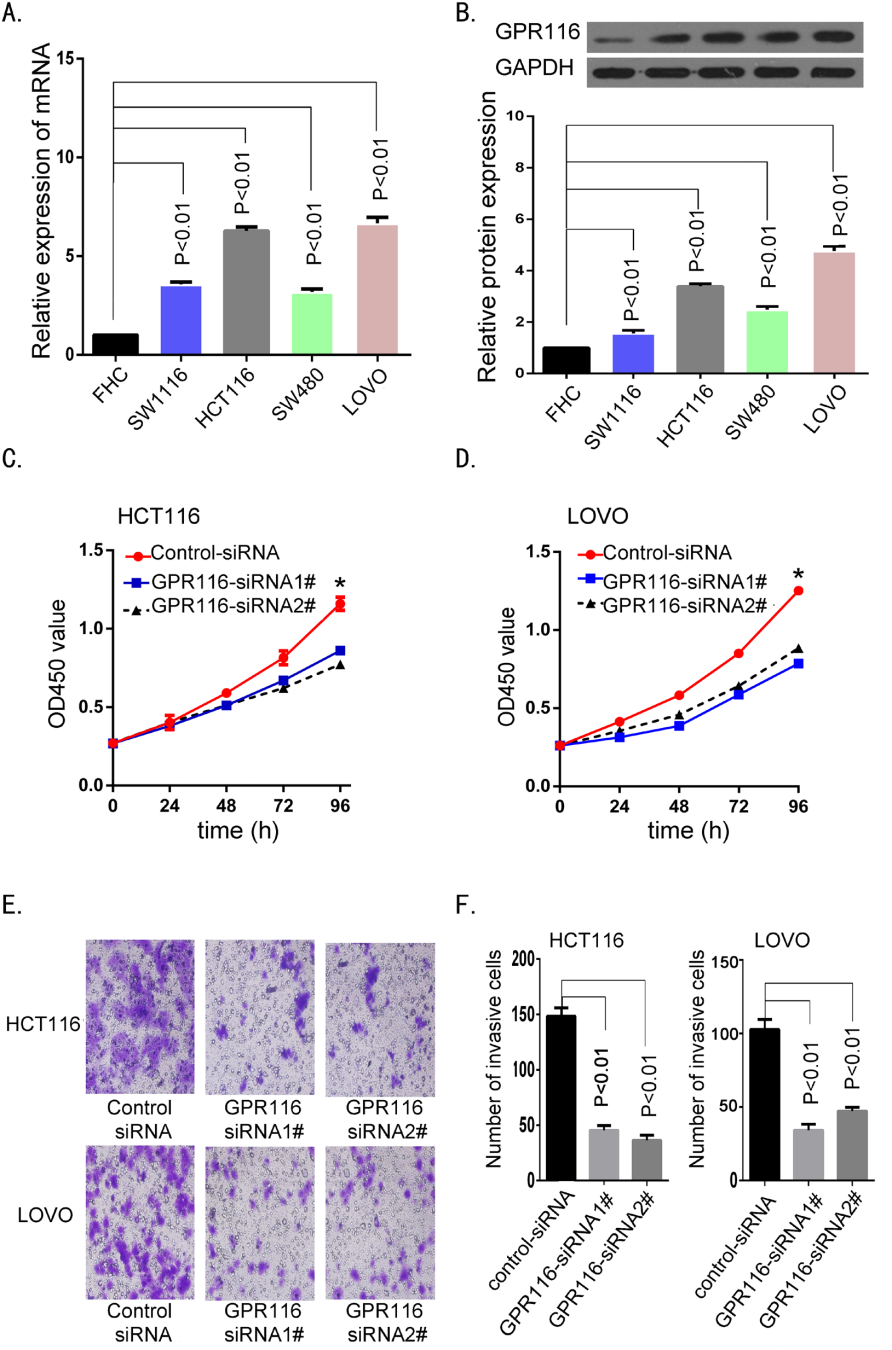
Downregulation of GPR116 inhibited colon cancer cell proliferation and invasion **(A-B)** GPR116 expression was measured in normal colonic cell line FHC and four colon cancer cell lines by real-time PCR and western bolt. **(C-D)** The cell viability was measured by CCK-8 assays at various time points in HCT116 and LOVO cell. (*p<0.05, versus control siRNA). **(E-F)** The cell invasion ability was measured by transwell chamber assay in HCT116 and LOVO cell; the results of transwell chamber assay were quantitated by counting invasive cells in five randomly selected high-power fields for three replicates (magnification, ×200). Results shown are the mean ± SD of triplicate determinations from three independent experiments.

To further verify whether GPR116 is involved in the development and progression of colon carcinoma, we firstly examined GPR116 expression in human colon cancer cell lines (HCT116, SW1116, SW480, LOVO) and one normal colonic mucosa epithelial cell line (FHC). The mRNA and protein expression of GPR116 were both significantly increased in CRC cell lines, especially in cell lines HCT116 and LOVO (*p*<0.01, Figure 6A-6B). Western blot analysis verified GPR116 protein was markedly downregulated in both HCT116 and LOVO cells transfected with siRNA-GPR116 1# and 2#. Secondly, biological function of GPR116 was evaluated using Cell Counting Kit 8 (CCK8) and transwell chamber assays. The CCK8 assay showed that knockdown of GPR116 greatly inhibited the proliferation ability of HCT116 and LOVO cells, compared with the control group (Figure 6C-6D). Transwell invasion assay demonstrated that the number of invasive cells was significantly reduced in the siRNA-GPR116-transfected groups (Figure 6E-6F). Altogether, these data revealed that as an oncogene, GPR116 promotes the proliferation and invasiveness of CRC cells.

### GPR116 knockdown suppressed EMT by AKT/ERK pathway

**Figure 7:**
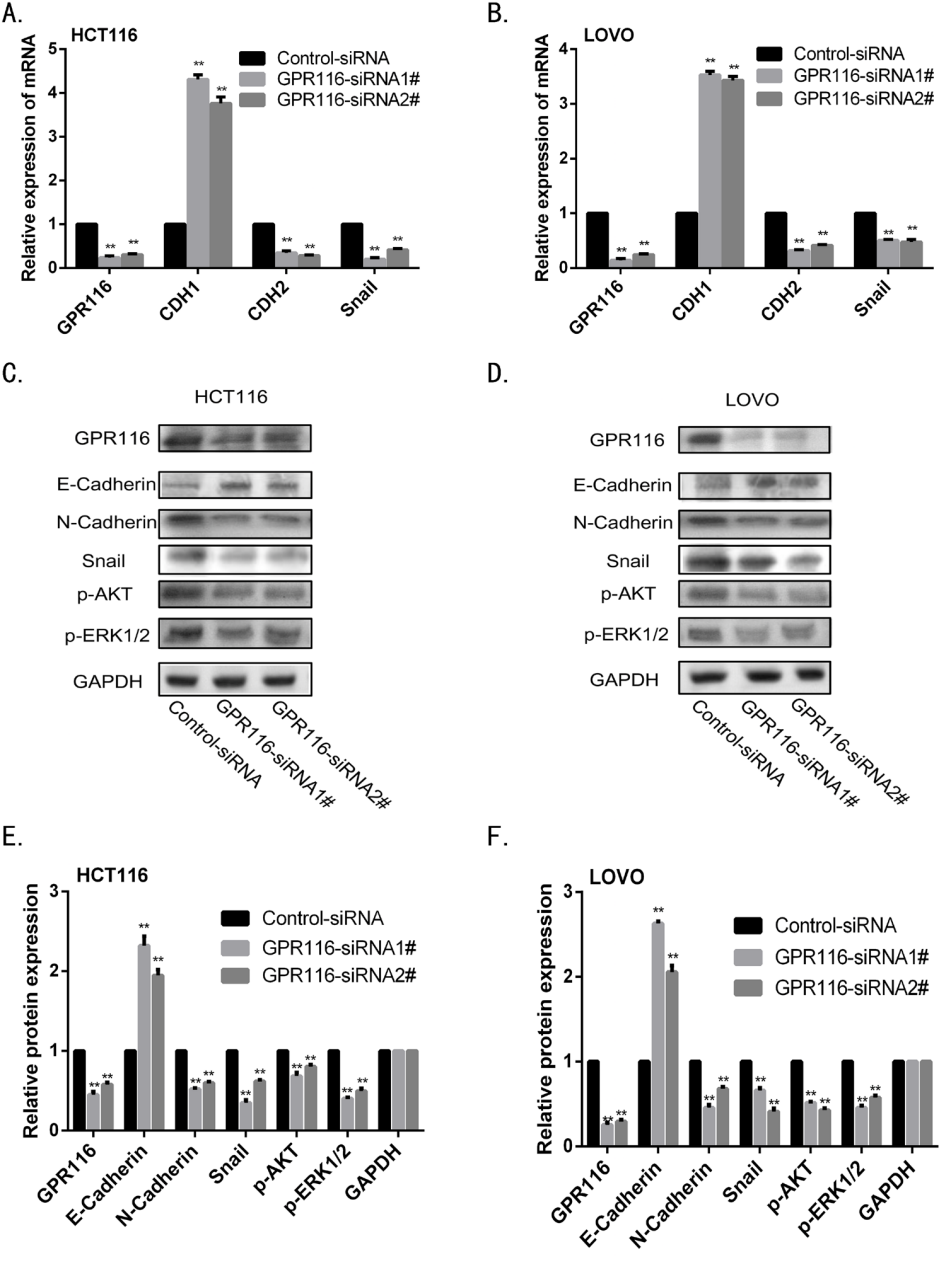
Downregulation of GPR116 reduces EMT through AKT/ERK pathway in CRC line **(A-B)** The mRNA level of epithelial markers (CDH1) and mesenchymal markers (CDH2 and Snail) were measured by real-time PCR in HCT116 and LOVO cell. **(C-F)** The protein level of epithelial markers (E-cadherin), mesenchymal markers (N-cadherin and Snail), p-AKT and p-ERK were measured by western blot in HCT116 and LOVO cell. Results shown are the mean±SD of triplicate determination from three independent experiments. **p<0.01, versus control siRNA.

Growing evidence has demonstrated EMT plays essential roles in tumor progression and metastasis. As shown in Figure 5C-5D, GSEA analysis revealed that “Gotzmann_Epithelial_To_Mesenchymal_Transiton_Up” and “Alonso_Metastasis_ EMT_Up” pathway were enriched in GPR116-high expression group compared with GPR116-low expression group. To confirm whether GPR116 regulate invasion through EMT processes in CRC, we explored the markers of EMT with RT-PCR and western blot. The results revealed that knockdown of GPR116 significantly increased the expression of E-cadherin, meanwhile reduced N-cadherin and Snail expression in HCT116 and LOVO cells transfected with siRNA-GPR116 compared with the control group (Figure 7A-7F). Recently, increasing evidence shows that AKT/ERK pathway is involved in EMT process. We surprisingly found that the expression of p-AKT and p-ERK1/2 were markedly reduced after knockdown of GPR116 (Figure 7C-7F), which indicates that the activity of AKT/ERK pathway was inhibited. Therefore, GPR116 may play a role of EMT progress by AKT/ERK signaling pathway in CRC. Further research are needed to illuminate its profound molecular mechanism of EMT.

## DISCUSSION

GPCR signaling plays a role in promotion of cell growth and survival, metastasis, and drug resistance [20]. GPCRs are prime targets for therapeutic intervention in cancers, accounting for 50% of all drug targets [21]. It was reported that GPCRs participated in the proliferation, migration and invasion of various cancers [19, 22–24]. Among these GPCRs, GPR116, a member of the adhesion sub-type of GPCRs family, was recently identified as a novel regulator of breast cancer metastasis [19].

Here, we firstly reported the role of GPR116 in colorectal carcinoma progression. Our study demonstrated that the expression of GPR116 was up-regulated in CRC tissues compared with corresponding adjacent non-cancerous tissues. Moreover, we found that increased GPR116 expression in CRC was correlated with histological differentiation and distant metastasis. Importantly, patients with higher GPR116 expression had significantly shorter survival time than those with lower GPR116 expression, which was also confirmed by GSE14333, GSE17536, and GSE33113 datasets. Cox regression analysis further demonstrated that GPR116 was an independent risk factor for poor prognosis of CRC patients.

As we know, metastasis is a leading cause of cancer-related death. When CRC patients were firstly diagnosed, some of them were found to have distant metastasis or vascular invasion, which might be result in unfavorable prognosis. EMT plays an important role in colon cancer invasion and metastasis [25, 26]. The first and crucial step of the EMT process is a local invasion through the epithelial basement membrane. The cells interact with other cells and cell matrix, resulting in dissociation from surrounding cells. The enhanced cell motility and decreased cell adhesion is a main step of cancer progression [27]. The characteristics of EMT manifest that epithelial markers decrease and mesenchymal markers increase. According to our clinical data and bioinformatics analyses above, we make a hypothesis that GPR116 may be involved in the invasion and metastasis process of CRC. To demonstrate this hypothesis, we performed transwell chamber assay and verified that the invasion ability of HCT116 and LOVO cells was remarkably reduced due to knockdown of GPR116. Besides, we further demonstrated that knockdown of GPR116 induced EMT by up-regulation of E-cadherin and down-regulation of N-cadherin and Snail in GPR116-depleted CRC cells. These results strongly suggested that GPR116 could contribute to CRC cell invasion and induce EMT.

More and more researches have verified that AKT/ERK signaling pathway was widely involved in EMT process [28]. Chuan et al. demonstrated that Gli-1 promoted colorectal cancer metastasis in a Foxm1-dependent manner by activating EMT and PI3K-AKT signaling pathway [29]. Hu et al. also verified that FAT1 prevented EMT via MAPK/ERK signaling pathway in esophageal squamous cell cancer [30]. Similarly with these researches, our study also revealed that knockdown of GPR116 obviously inhibited the protein expression of p-AKT and p-EKR1/2 in CRC cells. Therefore, we concluded that GPR116 may play a role of EMT process by AKT/ERK signaling pathway, resulting in the metastasis of CRC patients.

This study is our preliminary research about the role of GPR116 in CRC and detailed mechanisms remain to be elucidated. On the one hand, we are now carrying out further research to verify that GPR116 induce EMT by AKT/ERK signaling pathway both *in vitro* and *in vivo*. On the other hand, the relationship between GPR116 and the proliferation of solid tumors has not been studied. In this study, we detected GPR116 may contribute greatly to the CRC cell growth according to the results of CCK-8 assays and GSEA analysis. The profound molecular mechanisms of carcinogenesis and progression of GPR116 in CRC are needed to be explored in the future.

In conclusion, our study demonstrated that GPR116 was significantly upregulated in CRC tissues and could serve as an independent prognostic indicator for patients with CRC. Furthermore, our results provided evidence for GPR116 function as a novel inducer of EMT in CRC, which was at least partially through AKT/ERK signaling pathway. Therefore, we suggest that GPR116 could be considered as a risk factor and even a potential therapeutic target of CRC.

## MATERIALS AND METHODS

### Patients and tissue specimens

Human CRC tissue microarrays (TMA) including 90 pairs of primary CRC and corresponding noncancerous samples were collected in Renji hospital, affiliated to the Shanghai Jiaotong University, School of Medicine, between January 2010 and December 2010. Pathological information of the samples was retrieved from the pathology department of Renji Hospital. None of the patients had a history of other solid neoplasms, preoperative radiotherapy, chemotherapy and other anti-tumor treatments. The follow-up time was calculated from the date of surgery to CRC-related death, or the ultimate deadline (October 31, 2016). Additional Forty-eight paired fresh-frozen CRC specimens were collected from CRC patients with radical surgery in RenJi Hospital from June 2016 to October 2016, to test the mRNA and protein levels of GPR116 expression. Among these 48 patients, 42 patients's samples were collected for performing RT-PCR, and another 6 samples were collected for western blot.

This study was approved by the Ethics Committee of RenJi Hospital, School of Medicine, Shanghai JiaoTong University, and written informed consent was obtained from all the subjects.

### Bioinformatics analysis

The datasets in Oncomine Cancer Microarray database (https://www.oncomine.org) were used to determine the expression pattern of GPR116 in CRC. The dataset of corresponding clinical data used in this study were downloaded from the Gene Expression Omnibus (GEO). Data extraction was performed with R 3.0.2 software. Besides, a gene set enrichment analysis (GSEA) was performed to gain the biological functions and pathways involved in CRC pathogenesis through GPR116 pathway. The canonical pathways gene sets (c2.cp.v4.0.symbols.gmt) from the Molecular Signatures Database-MsigDB (http://www.broad.mit.edu/gsea/msigdb/index.jsp) were used for enrichment analysis. Only gene sets represented by at least 15 genes were retained [31].

### Cell culture and small interfering RNA silencing

Human colorectal carcinoma cell lines (HCT116, SW1116, SW480 and LOVO) and normal colonic mucosa epithelial cell line (FHC) were purchased from the American Type Culture Collection. They were cultured in RPMI 1640 medium (Gibco, Gaithersburg, MD, USA) with 10% FBS at 37°C in an atmosphere of 5% CO2.

Transfection of siRNA was performed using Lipofectamine 2000 (Invitrogen, USA) according to the manufacturer's protocol. The transfection reagent was replaced by complete medium after incubation for 6 h, and cells were harvested 24 h or 48 h later.

### Immunohistochemical (IHC) staining and scoring

Immunohistochemical staining was performed in 90 paired CRC patients and their corresponding normal colonic mucosa by tissue microarray (TMA). The TMA section was deparaffinated in xylene and rehydrated with different concentrations of alcohol. Then, the section was treated with 3% hydrogen peroxide, followed by retrieval antigen with 10 mM citrate buffer (pH 6.0) with microwave. After being blocked with 10% goat serum for 30 minutes, the section was incubated with rabbit GPR116 antibody (1:100, Proteintech Group, USA) overnight at 4°C, followed by a peroxidase-labeled secondary antibody.

The scoring was calculated based on the sum of the percentage of positive staining cancer cells (0%-5%: 0; 6%-35%: 1; 36%-70%: 2; >70%: 3). The scoring of staining intensity was also calculated (no staining: 0; weakly staining: 1; moderately staining: 2; strongly staining: 3). The final score was determined using the score based on the percent of positively stained cell × the score based on the staining intensity (0-1 score: “-”; 2-3 score: “+”; 4-6 score: “++”; score >6: “+++”). Low expression was defined as a total score <4, whereas high expression was defined as a total score >4. Two senior pathologists respectively and blindly calculated the score.

### Quantitative real-time PCR

Total RNA was extracted by Trizol reagent (Takara, Japan), and reversely transcribed through the PrimeScript RT-PCR kit (Takara, Japan) in accordance with the manufacturer's instructions. Quantitative real-time PCR was conducted with SYBR Premix Ex Taq (Takara, Japan) on a 7500 Real-time PCR system (Applied Biosystems, Inc., USA). Primer sequences used in this research were as follows: GPR116, forward 5’-CGGAAGGGTTACGGAATTTTACC-3’, reverse 5’-GTGATGGTGGTGTAGTCTTGAC-3’; E-Cadherin, forward 5’-AGAACGCATTGCCACA TACACTC-3’, reverse 5’-CATTCTGATCGGTTACCGT GATC-3’; N-Cadherin, forward 5’-GGAGACATTGG GGACTTCATT-3’, reverse 5’-TCCTGCTCACCA CCACTACTT -3’; Snail, forward 5’-TTACCTTCCA GCAGCCCTAC-3’, reverse 5’-GACAGAGTCCCAGAT GAGCA -3’; 18s RNA, forward 5’-CGGACAGGA TTGACAGATTGAT AGC -3’, reverse 5’-TGCCAGA GTCTCGTTCGTTATCG-3’. The 2^−ΔΔCt^ method was used to quantify the relative GPR116 expression levels and normalized to 18s RNA.

### Western blotting

Fresh-frozen tissues were lysed with RIPA (Beyotime, China) containing a protease inhibitor mixture (protease inhibitors; phosphatase inhibitors; PMSF; KangChen, Shanghai, China) on ice for 30 minutes. The concentration of protein was measured by a BCA protein assay kit (Pierce Biotechnology). Proteins were separated on 10% SDS-polyacrylamide gels and transferred on to polyvinylidene difluoride (PVDF) membranes (Millipore, Bedford, MA, USA). Then, the membranes were blocked with 5% nonfat milk and incubated with the primary antibodies at 4°C overnight. After that, the membranes were washed with TBST and incubated with HRP-labeled goat anti-rabbit antibody (1:5000, WeiAo, Shanghai, China) for 1 hour. Finally, the immunoreactive signals were detected using the ECL detection system (SuperSignal West Femto Maximum Sensitivity Substrate, Thermo Fisher Scientific, IL, USA). Sources of antibodies included rabbit anti-GPR116 (1:200, Proteintech Group, USA), rabbit anti-N-Cadherin (1:1000, Cell Signaling Technology, USA), rabbit anti-E-Cadherin (1:1000, Cell Signaling Technology, USA), rabbit anti-Snail (1:1000, Cell Signaling Technology, USA), rabbit anti-p-AKT (Ser473) (1:2000, Cell Signaling Technology, USA) and rabbit anti-p-ERK1/2 (Thr202/Tyr204) (1:2000, Cell Signaling Technology, USA), GAPDH (1:3000, KangChen, Shanghai, China).

### Cell viability assays

Cell viability was assessed by a tetrazolium salt (WST-8)-based colorimetric assay provided by the Cell Counting Kit 8 (CCK-8, Dojindo, Kumamoto, Japan). Treated and untreated CRC cells (5×10^3^ cells/well) were seeded into 96-well plates. At specified time points, 100 μl of CCK-8 solution (diluted in 1:10 with serum-free medium) was added to each well and the plates were incubated for 2 h. Cell viability was determined from absorbance readings at 450 nm.

### Transwell invasion assays

Cell invasion assays were performed on transwell chamber with 8μm pore-size filters with coated with Matrigel on the upper side (BD Biosciences). CRC cells with different treatment were then harvested, and 1×10^5^ cells were seeded in serum-free medium into the upper chamber, whereas medium supplemented with 20% fetal bovine serum was applied to the lower chamber as a chemoattractant to induce invasion. At the end of the assay, invaded cells on the bottom surface of the filter were fixed, stained and counted. The number of cells from five random microscope per filter was counted under an inverted microscope at 200 ×magnification.

### Statistical analysis

The statistical analysis and the graphical representations were performed respectively by SPSS 17.0 software (SPSS Inc, Chicago, IL, USA) and GraphPad Prism 6.0 software (San Diego, CA). Correlation of GPR116 expression with clinicopathologic factors in patients with CRC was evaluated by Chi-square test. Survival curves were generated using the Kaplan–Meier method. The differences between survival curves were calculated by the Log-rank test. The significant impact of parameters on survival was analyzed by Cox proportional hazards model using univariate and multivariate Cox regression analysis. The comparison between two groups was evaluated by Student's t-test. *p*<0.05 was considered statistically significant.
